# A Patient Presenting with Lower Extremity Paralysis due to Acute Aortic Occlusion

**DOI:** 10.1155/2022/9217012

**Published:** 2022-03-14

**Authors:** Theodore Strom, Mark McIntosh

**Affiliations:** Department of Emergency Medicine, UF Health Jacksonville, Florida, USA

## Abstract

Acute aortic occlusion (AAO) is a rare and life-threatening condition that is rarely described in limited case series over the past several decades. The etiology and management are diverse across documented accounts, but prompt recognition facilitated by performing a thorough vascular and neurologic exam is critical to prevent delayed diagnosis and adverse outcomes. We report a patient who presented to the emergency department with the complaint of acute-onset lower extremity paralysis due to acute aortic occlusion. Her condition was rapidly diagnosed with a CT angiogram protocolized for aortic dissection and managed with anticoagulation and thrombectomy with eventual near complete recovery of her lower extremity function.

## 1. Introduction

Acute aortic occlusion (AAO) is a rare pathology that has a high rate of morbidity and mortality which requires prompt diagnosis and treatment. Barring trauma as a risk factor, the literature reports thrombus formation resulting from acute embolic occlusion or local formation. In documented cases of AAO secondary to embolization, previous studies have identified etiologies that include left ventricular thrombus, valvular heart disease, thoracic mural thrombus, and cardiac myxoma [1–3]. AAO caused by thrombotic processes often evolves following long-standing structural pathology to the aorta. This may include the formation of an abdominal aortic aneurysm, atherosclerotic changes, or the presence of hypercoagulable states. The studies have identified antiphospholipid antibody-induced thrombocytopenia, heparin-induced thrombocytopenia, protein C deficiency, and more recently infection with COVID-19 as likely hypercoagulable culprits [1–5]. A more recent trend in the literature suggests a higher prevalence of thrombotic over embolic etiologies. This is hypothesized to be the result of more aggressive anticoagulant treatment of proembolic disorders such as atrial fibrillation [2, 6].

The clinical presentation of AAO can be as dynamic as the underlying pathogenesis. It usually develops in older patients at a mean age of 69 years, often with cardiac comorbidities such as congestive heart failure, coronary artery disease, hypertension, hyperlipidemia, diabetes, peripheral vascular disease, and arrhythmia [1, 4, 6]. Patients exhibit a variety of clinical features including extremity mottling, cauda equina syndrome, paraplegia, acute abdomen, hypothermia, and limb ischemia [1, 2, 7, 8]. A timely diagnosis requires recognition of these characteristics as a primary vascular syndrome rather than a primary spinal cord pathology presenting symptoms of anterior cord compression. Any delay in diagnosis can have severe consequences, as primary treatment often requires emergent revascularization using an open surgical or endovascular technique. The following case involves a patient with few underlying health conditions who presented symptoms related to spinal cord compression with the ultimate diagnosis of acute aortic occlusion.

## 2. Case Presentation

**Figure 1:**
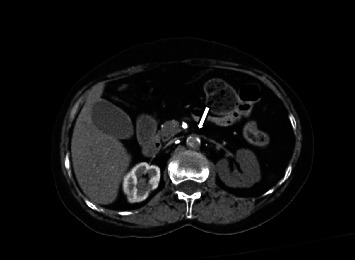
CT angiogram demonstrating AAO (arrow) with hypoperfusion of the left kidney.

The patient is a 58-year-old woman with a medical history of hypertension, depression, and remote intravenous drug use who came to the emergency department for acute-onset paralysis involving bilateral lower extremities, urinary retention, and abdominal pain. Before the onset of symptoms, the patient reported experiencing a flu-like syndrome (prior to the COVID-19 pandemic). She denied having a history of vascular or heart disease and reported no recent use of IV drugs. The initial physical exam showed sinus tachycardia without hypotension, absent rectal tone, mottled appearance in the lower extremities, and absence of bilateral femoral pulses. A CT angiogram protocolized for aortic dissection demonstrated complete occlusion of the abdominal aorta from the level of the left renal artery with near complete hypoperfusion of the left kidney and adrenal gland (Figure 1). The occlusion extended to the bifurcation of the bilateral external and internal iliac arteries.

The patient received unfractionated heparin and underwent emergent thrombectomy by interventional vascular radiology followed by bilateral iliac catheter-directed thrombolysis. The patient's ICU course was complicated by postperfusion rhabdomyolysis and renal failure that required temporary hemodialysis. An echocardiogram revealed a mobile, pedunculated apical thrombus in the left ventricle measuring 2.5 × 1.3 cm that resolved with anticoagulation therapy after revascularization. During the hospital course, an oral anticoagulant was initiated and lifelong anticoagulation recommended. At the time of discharge, the patient had regained most of her lower extremity strength only demonstrating weakness with dorsiflexion of her right foot that required further home physical therapy.

## 3. Discussion

Sudden occlusion of the aorta is a life-threatening condition. Data gathered in recent series have estimated an incidence of 3.6 per 1,000,000 individuals annually with a mortality ranging from 25% to 75% [2, 4, 6, 7]. Therefore, early recognition of signs and symptoms of AAO leading to diagnostic accuracy and intervention is critical to achieve the best clinical outcome in this high-risk condition. Our patient presented with a medical history which was not suggestive of an increased risk for developing a cardiac or aortic source of thrombus formation other than a history of a recent flu-like syndrome and remote IV drug use. However, a COVID screening test would be warranted in the present practice environment recognizing that a recent COVID-19 infection is associated with coagulation abnormalities that create a hypercoagulable state. Despite extensive evaluation, no other sources for the cardiac thrombus were identified including evidence of endocarditis or valvular heart disease, cardiomyopathy, atrial fibrillation, patent foramen ovale, or an underlying genetic or acquired cause of hypercoagulability.

Unfortunately, there is often a major challenge in recognizing AAO in a timely manner. As in our patient, a large infrarenal aortic thrombus led to an occlusion of the anterior spinal artery (artery of Adamkiewicz). Patients suffering an AAO have a spectrum of clinical features, including the isolated complaint of sudden-onset paraplegia. The clinician must be diligent in the initial evaluation to perform a thorough vascular exam to avoid the consequences of a delayed diagnosis while pursuing a primary spinal cord pathology [1, 2, 4, 6–8]. Fortunately, many cases of AAO with an evolving neurologic deficit experience partial or complete recovery after timely revascularization [1, 7, 8]. This supports the research using animal models that demonstrate a reduction in the time of spinal cord ischemia is correlated with an improved neurologic outcome and reduced overall morbidity and mortality [8].

## 4. Conclusion

Acute aortic occlusion is a condition with potential for high morbidity and mortality, but complications can be mitigated when promptly recognized. A comprehensive history and exam that includes a thorough vascular assessment can greatly assist the clinician in rapid recognition of the underlying pathology leading to appropriate medical and surgical interventions.

## Data Availability

No data were used to support this study.
